# Isocyanate exposure and asthma in the UK vehicle repair industry

**DOI:** 10.1093/occmed/kqv108

**Published:** 2015-07-25

**Authors:** S. J. Stocks, K. Jones, M. Piney, R. M. Agius

**Affiliations:** ^1^NIHR Greater Manchester Primary Care Patient Safety Translational Research Centre, Centre for Primary Care, Institute of Population Health, University of Manchester, Manchester M13 9PL, UK,; ^2^Health & Safety Laboratory, Buxton, Derbyshire SK17 9JN, UK,; ^3^Health & Safety Executive, Bootle, Merseyside L20 7HS, UK,; ^4^Centre for Occupational and Environmental Health, Centre for Epidemiology, Institute of Population Health, University of Manchester, Manchester M13 9PL, UK.

**Keywords:** Biological monitoring, HDI, hexamethylene diisocyanate, isocyanate exposure, motor vehicle repair, occupational asthma, trends, two-pack spray paints, work-related asthma.

## Abstract

**Background:**

Organic diisocyanates are a common cause of occupational asthma, particularly in motor vehicle repair (MVR) workers. The UK Health & Safety Laboratory provides screening for urinary hexamethylenediamine (UHDA), a biomarker of exposure to 1,6-hexamethylene diisocyanate (HDI). The UK Surveillance of Work-related and Occupational Respiratory Disease scheme (SWORD) has collected reports of occupational asthma since 1996.

**Aims:**

To compare trends in HDI exposure with trends in the incidence of work-related asthma attributed to isocyanates or paint spraying in MVR workers reported to SWORD.

**Methods:**

Two-level regression models were used to estimate trends in UHDA levels and work-related asthma in MVR workers reported to SWORD. The direction and magnitude of the trends were compared descriptively.

**Results:**

From 2006 to 2014, there was a significant decline in the number of urine samples with detectable levels of UHDA (odds ratio = 0.96; 95% confidence intervals 0.94–0.98) and minimal change in those over the guidance value (1.03; 1.00–1.06). Over the same period, there was a significant decline in all asthma cases attributed to isocyanates or paint spraying reported to SWORD (0.90; 0.86–0.94) and a non-significant decline among MVR workers (0.94; 0.86–1.02).

**Conclusions:**

The simultaneous decrease in HDI exposure and incident cases of asthma reported to SWORD is temporally consistent with a reduction in exposure to airborne isocyanate leading to a reduction in asthma. Although this is not direct evidence of a causal relationship between the two trends, it is suggestive.

## Introduction

Organic diisocyanates are respiratory and skin sensitizers that have been a longstanding common cause of occupational asthma in the UK [1]. Varnishes, coatings and two-pack spray paints used in body shops, particularly in the motor vehicle repair (MVR) industry, commonly contain the organic aliphatic diisocyanates, 1,6-hexamethylene diisocyanate (HDI) and isophorone diisocyanate [2]. All workers exposed to airborne isocyanates should have appropriate health surveillance unless a risk assessment indicates there is little or no risk to their health [3]. The Health & Safety Laboratory (HSL) in the UK has developed a method to measure urinary levels of hexamethylenediamine (HDA), a metabolite of HDI, for monitoring workers’ exposure to isocyanates [4] and was the sole UK commercial provider of urinary HDA (UHDA) monitoring from 1996 to 2011. The current guidance value for UHDA, derived from the 90th percentile of biological monitoring data from workplaces with exposure to isocyanates, is 1 µmol UHDA/mol creatinine [2]. Exceeding this value should trigger an investigation into the workplace exposure controls.

From 2004 to 2008, the Health & Safety Executive, in collaboration with industry and other stakeholders, ran a national body shop project which aimed to reduce exposure to isocyanates in MVR. Safety and Health Awareness Days (SHADs) provided information about asthma and advice about clearance times before entering the spray painting booth without personal protective equipment. Free UHDA analysis was offered to SHADs attendees. Those employers declining to participate were more likely to be inspected during the lifetime of the project. In addition, new guidance was written in association with, and supported by, the spray booth and paint manufacturers and SHAD material was supplied free of charge to training colleges and trade associations. From October 2007, a topic-based inspection pack was used to guide inspectors visiting MVR premises [5]. All body shops inspected were offered free UHDA analysis. Following the project, UHDA levels were found to be lower in SHADs attendees and in screening samples collected after the project [6]. At the same time, there was a non-significant decline in occupational asthma and short latency respiratory disease in MVR workers relative to other industries reported to the Surveillance of Work-related and Occupational Respiratory Disease (SWORD) scheme, a UK-based occupational respiratory disease surveillance network [7].

Our hypothesis is that trends in exposure to HDI, as reflected in UHDA samples submitted to HSL, will be consistent with changes in the incidence of asthma attributed to spray painting or HDI in MVR workers. Furthermore, we aimed to investigate whether the reductions in exposure and asthma incidence observed previously during the national body shop project had been maintained following the end of the project. To do this, we compared trends in the number of workers employed in MVR with detectable UHDA levels or levels over the guidance limit, with trends in incidence of asthma in MVR workers reported to SWORD.

## Methods

Samples submitted to HSL through routine screening, workplace inspections or the SHADs from workers in MVR, coachworks or boat, trailer, caravan and aircraft repair industries between 2006 and 2014 were included in the analysis. Workers were asked to provide urine samples post-shift, or post-exposure if they sprayed intermittently. Samples were analysed for HDA by gas chromatography–mass spectrometry following acid hydrolysis as described previously [6]. If workers had more than one result on the same day, only the highest value was included in the analysis. The samples were categorized in to two binary variables: (i) HDA detected (UHDA > 5 nmol/l; results were creatinine-corrected to adjust for urinary dilution) and (ii) HDA exceeding the UK guidance value (1 µmol/mol creatinine).

The SWORD scheme has been described in detail elsewhere [8]. Briefly, reports of asthma were returned to SWORD by respiratory physicians. Some physicians reported every month, others during one randomly selected month per year. If no cases were seen, a zero case report should have been made. Physicians reported cases which in their opinion, on a balance of probabilities, had been caused or aggravated by work and also reported the patient’s occupation and suspected causal agent. Cases of asthma attributed to spray painting or isocyanates in workers in the industries and occupations as listed above were analysed.

The time trends in the two types of data described above, case counts of asthma and a binary variable according the level of UHDA, were estimated. A negative binomial regression model with year as the main predictor of interest was used to analyse case counts of asthma and a corresponding logistic regression model was used for the UHDA variable. For both types of data, a two-level version of the model was used with the SWORD reporting centre as a ‘random effect’ for the asthma case counts and the company for the UHDA variable. The advantage of the two-level regression model is that it can estimate ‘within-centre’ or ‘within-company’ changes over time and is not affected by changes over time in the number of centres or companies itself. Furthermore, it allows for between-centre or between-company variation in incidence producing more accurate *P* values and confidence intervals (CIs) than the simple one-level model.

Specifically for the UHDA analysis, the binary UHDA variables were the outcome measure in a logistic regression model with year as the main predictor and seasonal variation as a covariate with random effects at the company level. However, some samples could not be linked back to the original company (the random effects variable) meaning that they did not fit within the structure required for the two-level model; therefore, a standard, one-level, logistic regression model including all samples is also reported. A further covariate that distinguished between samples arising from routine screening, SHADs or inspections was included, thereby allowing the trends to vary within each category. The results are expressed as odds ratios (ORs) reflecting changes in the number of workers exposed at the defined level relative to a reference time period (2006–08) or an individual year (2008).

For the asthma case counts, a two-level negative binomial model with β distributed random effects was used. The outcome measure was case counts and the main predictor was year. The model was also adjusted for seasonal variation (month), type of reporter (monthly or 1 month per year) and first month as a new reporter as described previously [9]. The results are expressed as incidence rate ratios reflecting changes in the incidence of asthma within reporters relative to an individual year (2008) or a reference time period (2006–08). For the latter, the cases were aggregated into 3 year periods due to the low numbers of reported cases within specific occupational groups. Ethical approval for SWORD was obtained from NRES Committee North West—11/NW/0832.

## Results

**Figure 1.**
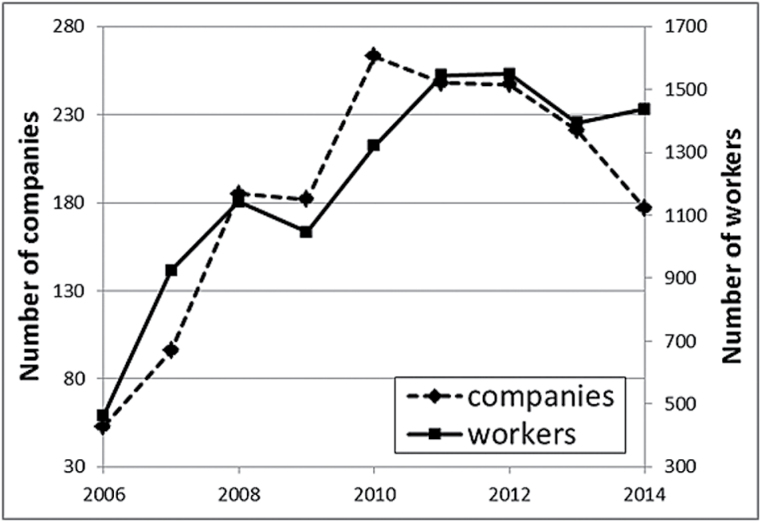
Numbers of workers and companies in MVR, coachworks or boat, trailer, caravan and aircraft repair industries providing samples to HSL for UHDA analysis.

From 2006 to 2014, there were 16352 UHDA results recorded for workers in MVR, coachworks or boat, trailer, caravan and aircraft repair industries. After excluding 1346 repeat results on the same date and retaining the highest value, 15006 (92%) were included in the analysis. From 2006 to 2010, the number of companies submitting samples for screening, either directly or through the SHADS programme, increased from 53 to 263 and then decreased from 2011 when HSL ceased to be the sole analysis provider (Figure 1). The mean number of workers tested per company per year was 6.9. Of the 13317 screening samples, 10036 (75%) came directly from the company and 3281 (25%) came via an occupational health provider. Assuming that the companies being screened by occupational health providers were similar in size to those submitting their own samples, ~1140 companies had their workers screened at least once between 2006 and 2014. There are an estimated 8000 body shops in the UK, suggesting that ~14% of these participated in screening from 2006 to 2014, in agreement with previous estimates [10]. The distribution of the samples according to industry and the reason for collection is shown in Table 1.

**Table 1.** Results of UHDA analysis by type of sample and industry of origin

Industry providing sample	UHDA level	No. exceeding specified UHDA level tests/all tests (%)		
		Screening	SHADs	Inspection
MVR	Detected	1937/9935 (19)	123/815 (15)	139/714 (19)
	>1^a^	687/9935 (7)	47/815 (6)	53/714 (7)
Coachworks, trailers, caravans, aircraft and boat repair	Detected	612/3382 (18)	–	32/160 (20)
	>1^a^	256/3382 (8)	–	20/160 (13)

^a^UHDA level > 1 µmol/mol creatinine.

There was no significant difference between MVR workers and workers employed in coachworks or boat, trailer, caravan and aircraft repair in levels of urinary HDA over the guidance value (6.9% versus 7.8%) or the number of samples with UHDA detected (19.2% versus 18.2%). There were also no significant differences between the source of the sample (screening, SHADs or inspection) and the number of workers with UHDA exceeding the guidance value, but samples originating from the SHADs were significantly less likely to have detectable UHDA than the screening or inspection samples (*P* < 0.05).

From 2002 to 2014, there were 223 reports to SWORD of asthma attributed to isocyanate exposure, of which 88 also had spray painting specified as the isocyanate source, plus 12 reports of asthma attributed to spray painting without specifying isocyanate exposure. Of the 235 reports attributed to isocyanates or spray painting, 82 (35%) were workers in MVR, coachworks or boat, trailer, caravan and aircraft repair industries and 60 (26%) of these worked in MVR.

**Figure 2.**
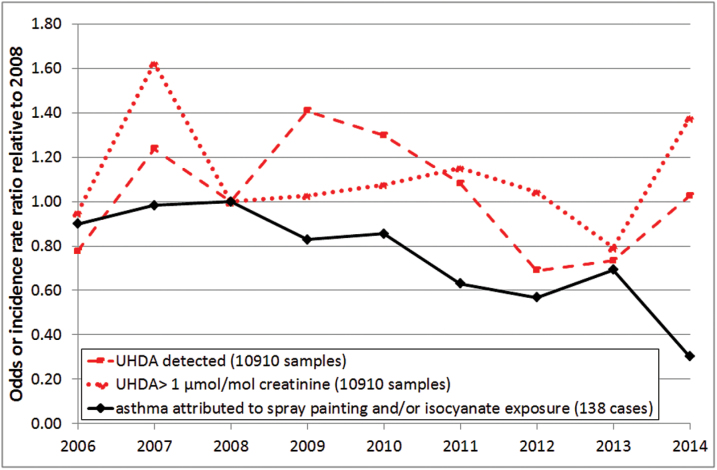
Comparison of annual changes in the number of workers with UHDA detected or over the guidance threshold (two-level logistic regression model OR relative to 2008) with changes in the incidence of asthma reported to SWORD (two-level negative binomial model incidence rate ratio relative to 2008).

**Figure 3.**
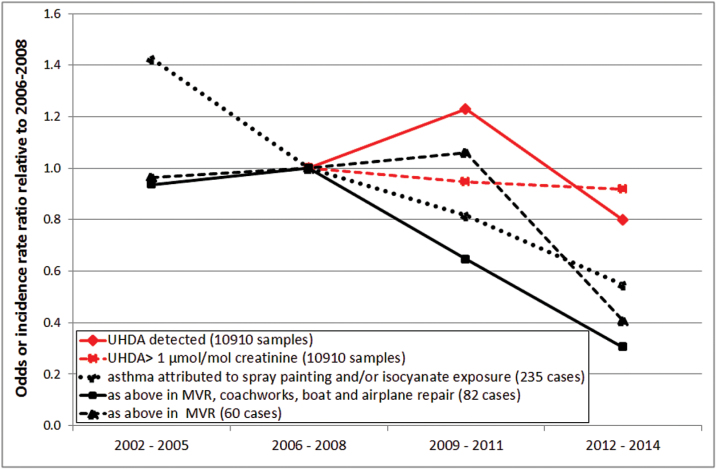
Comparison of 3 year aggregates of changes in the number of workers with UHDA detected or over the guidance threshold (two-level logistic regression model OR relative to 2006–08) with changes in the incidence of asthma reported to SWORD (incidence rate ratio relative to 2006–08).

As not all samples could be linked back to the original company, two types of model were used for the analysis. A standard (one level) logistic regression model included all samples (*n* = 15006) and a two-level logistic regression model included all screening samples submitted directly by the companies and the inspection samples (*n* = 10910). Overall, there was no significant change in the number of samples over the guidance limit from 2006 to 2014 (two-level logistic regression model: OR = 1.00, CI 0.96–1.04 and standard logistic regression model: OR = 1.03, CI 1.00–1.06) but within that period, there was an increase between 2006 and 2007 followed by a decline until 2013 and an increase during 2014 (Figure 2). Over the same period, there was a significant decline in the number of samples with detectable levels of urinary HDA (two-level logistic regression model: OR = 0.96, CI 0.93–0.99 and standard logistic regression model: OR = 0.96, CI 0.94–0.98) and a similar pattern to that described above, except that the increase continued up to 2009. Both models showed the same pattern but only the two-level logistic regression model results are shown in Figure 2 for clarity. There were no significant differences between the trends depending upon the source of the sample (screening, inspections or SHADs). Over the same time period, there was a decline in incidence of asthma attributed to isocyanates and paint spraying in all industries (0.90, 0.86–0.94; Figure 2), spray painting in all industries (0.94, 0.88–1.00) and isocyanates or paint spraying in MVR (0.94, 0.86–1.02). As the numbers of reports of asthma from the MVR industry were low, cases were aggregated into 3 year periods for comparison with changes in HDI exposure over the same time period (Table 2; Figure 3). While the incidence of asthma attributed to paint spraying and isocyanates in all industries was declining from 2002, the incidence in MVR and other body shop industries did not start to decline until after 2008, coinciding with the SHADs and increasing uptake of UHDA screening. Although the number of workers with detectable levels of UHDA increased between 2009 and 2011, there was no corresponding increase in asthma in MVR workers, more in keeping with the decrease in the number of workers with UHDA above the guidance value over the same period.

**Table 2.** Changes (relative to 2006–08) in the number of workers with UHDA above the guidance value and the incidence of asthma

Time period	MVR, coachworks or boat, trailer, caravan and aircraft repair		MVR
	UHDA, OR (95% CI), samples = 10897	Asthma, IRR (95% CI), cases = 82	Asthma, IRR (95% CI), cases = 60
2002–05	−	0.94 (0.54–1.61)	0.97 (0.47–1.31)
2006–08	1.00	1.00	1.00
2009–11	0.86 (0.67–1.10)	0.65 (0.34–1.22)	1.06 (0.34–1.08)
2012–14	0.81 (0.63–1.06)	0.31 (0.12–0.75)	0.41 (0.24–0.90)

IRR, incidence rate ratio.

## Discussion

We observed a decrease in the number of workers with exposure to HDI in MVR that coincided with a decrease in the incidence of asthma attributed to paint spraying and isocyanates in MVR workers reported to SWORD. Furthermore, the incidence of asthma attributed to isocyanates or paint spraying in all industries was declining from 2002, whereas the incidence in MVR and other relevant industries began to decline after 2008, i.e. after the introduction of UHDA screening and the national MVR body shop project. However, there appears to be an increase between 2013 and 2014 in both the number of workers with UHDA detected and those over the guidance limit. There is no corresponding increase in the incidence of asthma but we might expect a lag between increasing levels of exposure and increasing incidence of asthma. The implication is that the impact of the national body shop project might be fading. The lower numbers of samples with detectable levels of UHDA submitted through the SHADs suggests that this intervention was effective and that a refresher intervention might be beneficial.

Although the decrease in exposure is temporally consistent with the decrease in asthma, we do not have direct evidence for a causal relationship. The question remains whether the samples returned to HSL and cases reported to SWORD are nationally representative. In 2005, ~70% of eligible physicians reported to SWORD. As body shops are distributed throughout the country, it is unlikely that any regional bias would affect the number of cases in MVR workers reported to SWORD. There is a greater possibility of bias in the UHDA samples as businesses with poorer health and safety practices might choose not to send samples for screening. However, these businesses might be encouraged to submit samples for UHDA analysis at inspections. The increase in uptake of routine screening from 2006 to 2011 is encouraging although it appears that the majority of UK body shops are still not participating in UHDA screening. The decline in samples sent to HSL in 2011 reflects other providers of UHDA screening entering the market and not necessarily a decrease in screening uptake.

The structure of both data collections means that individual reporters (physicians or companies) will enter or leave the reporting cohort over time and this was considered in the two-level regression models by including random effects on the reporting physician or company. It is particularly helpful in allowing for the fact that HSL ceased to be the sole provider of HDA analysis from 2011 by allowing the trend to vary within companies. As not all samples could be traced back to their original company not all samples could be included in the two-level model but the standard regression model using all samples gave similar results, increasing the confidence in the results. Furthermore, the model was adjusted for the source of the sample thereby allowing the trend for each type of sample (screening, SHADs or inspection) to vary independently.

The higher proportion of samples exceeding the guidance value in workers employed in coachworks or boat, trailer, caravan and aircraft repair might be related to spraying overhead due to the larger items being sprayed, as observed previously [4].

Overall, we report a simultaneous decrease in both the number of MVR workers with detectable levels of UHDA and those exceeding the guidance value, and incident cases of work-related asthma consistent with a reduction in occupational exposure to isocyanates leading to a reduction in work-related asthma attributed to isocyanates or paint spraying. The increase in the number of workers with a UHDA level above the guidance value observed during 2014 suggests that the declining trend in exposure may not be sustainable without further intervention.

**Key points**

- Isocyanate exposure was a common cause of asthma in the UK motor vehicle repair industry and workers potentially exposed to 1,6-hexa methylene diisocyanate can be screened for urinary hexamethylenediamine, a biomarker of exposure.
- A declining trend in the number of workers with detectable urinary hexamethylenediamine levels measured during screening temporally coincided with a declining trend in incidence of asthma in the motor vehicle repair industry reported to the Surveillance of Work-related and Occupational Respiratory Disease occupational respiratory disease surveillance scheme from 2006 to 2014.
- The increase in the number of workers with a urinary hexamethylenediamine level above the guidance value observed during 2014 suggests the need for continuing vigilance by the industry and that the declining trend in exposure may not be sustainable without further intervention.

## Funding

SWORD is funded by the Health & Safety Executive (HSE) in Great Britain (grant no. JN4243).

## Disclaimer

Any opinions and/or conclusions expressed are those of the authors alone and do not necessarily reflect HSE policy.

## Conflicts of interest

None declared.
